# Influence of Cementitious System Composition on the Retarding Effects of Borax and Zinc Oxide

**DOI:** 10.3390/ma12152340

**Published:** 2019-07-24

**Authors:** Feraidon F. Ataie

**Affiliations:** Concrete Industry Management Program, California State University, Chico, CA 95929, USA; fataie@csuchico.edu

**Keywords:** zinc oxide, borax, set time, heat of hydration, calcium aluminate cement, calcium sulfoaluminate cement

## Abstract

This research investigated the retarding impact of zinc oxide (ZnO) and borax (Na_2_[B_4_O_5_(OH)_4_]·8H_2_O) on hydration of Portland cement, calcium aluminate cement (CAC), and calcium sulfoaluminate cement (CSA). Heat of hydration of cement paste samples with and without ZnO and borax was used to measure the influence of ZnO and borax on the set time of these cementitious systems. It was found that both ZnO and borax can retard the set time of Portland cement systems; however, ZnO was shown to be a stronger set time retarder than borax for these systems. ZnO did not show any retarding impact on CAC and CSA systems while addition of borax in these systems prolonged the set time. It was concluded that ZnO does not poison the nucleation and/or growth of CSA and CAC hydration products. We suggest that borax retards the cement set time by suppressing the dissolution of cement phases.

## 1. Introduction

Utilization of cement set time retarders, referred to as retarding admixtures, in concrete allows concrete producers to delay the set time of concrete. Retarding admixtures are commonly used in hot weather concreting and when ready mixed concrete should be transported for a long distance. Sucrose (sugar) is a well-known cement set retarder [1,2,3,4,5]. Sucrose retards cement hydration by poisoning nucleation sites for calcium silicate hydrate (C–S–H) [3,6]. This poisoning effect prevents the formation of C–S–H for an extended period and thus delays the cement set time. The following abbreviations will be used in this paper: H = H_2_O, C = CaO, A = Al_2_O_3_, Ŝ = SO_3_, S = SiO_2_.

Zinc oxide (ZnO) has been shown to be another strong cement set retarder [7,8,9]. It has been suggested that ZnO, like sucrose, poisons C–S–H nucleation sites and thus retards the cement set time [7]. In cementitious systems containing ZnO, during the prolonged dormant period, cement particles continue to dissolve and thus the concentration of ions in the pore solution increases [3,7]. This will increase calcium concentration in the system leading to an increase in C–S–H nucleation sites. Therefore, the rate at which C–S–H grows after the set time is higher in systems containing ZnO compared to those without any retarder. This is why ZnO is called a “delayed accelerator” [7].

The retardation time of cementitious systems containing ZnO is correlated to the amount of Zn ions dissolved in the pore solution [7]. The higher the Zn ion concentration in the pore solution, the longer the retardation period. The retardation period caused by ZnO is suggested to end by two possible mechanisms [7]: (1) removal of Zn ions from the pore solution by chelation or adsorption of Zn ions by hydration products, such as C–S–H; and (2) removal of Zn ions by formation of calcium zinc hydrate according to Equations (1) and (2) [10,11].

(1) $$ZnO+H_{2}O+2OH^{-}\to \;Zn{\left(OH\right)}_{4}^{2-}$$

(2) $$Zn{\left(OH\right)}_{4}^{2-}+Ca^{++}+H_{2}O\to \;Ca{\left(Zn{\left(OH\right)}_{3}\right)}_{2}.2H_{2}O+2OH^{-}$$

Borax is another set time retarder for cementitious systems [12]. It has been shown that borax delays the set time of calcium sulfoaluminate cement (CSA) by preventing the dissolution of ye’elimite [12].

The behavior of retarding admixtures in cementitious systems depends on several factors, such as the composition of the systems as well as the curing temperature. Addition of supplementary cementitious materials (SCM), such as rice straw ash and silica fume, in cementitious systems have been shown to reduce the retarding action of chemical retarders [7,13]. This has been attributed to existence of more C–S–H nucleation sites in systems containing SCMs compared to those without SCMs [7,13,14]. Therefore, the composition of the cementitious system affects the chemical admixture performance in the system.

The dissolution of ZnO in alkaline solutions (such as cementitious solutions) increases as the temperature increases [15]. Similarly, cement early hydration, and thus the formation of C–S–H, increases as the curing temperature increases [16]. It is not known, however, as to how the curing temperature could affect the retarding action of ZnO on cementitious systems.

Although the impact of ZnO on Portland cement hydration has been studied by some researchers, the influence of ZnO on hydration of calcium aluminate cement (CAC) and on CSA has not been investigated yet. As the composition of CAC and CSA and their hydration mechanisms are different than that of Portland cement, it would be expected that ZnO and borax would affect the hydration of CAC, CSA, and Portland cement in different ways.

This study investigates the impact of ZnO and borax on the Portland cement, CAC, and CSA hydration process. Furthermore, the influence of the curing temperature on the retarding action of ZnO is studied.

## 2. Materials and Methods

ASTM C150 [17] Type II/V and Type III as well as commercially available CAC and CSA cements were used in this study. The chemical composition of the cementitious materials is shown in Table 1. ACS grade zinc oxide was used. The borax used was ACS grade sodium tetraborate decahydrate (Na_2_[B_4_O_5_(OH)_4_]·8H_2_O). Fifty percent food grade citric acid solution was used to stabilize the set time of the CSA cement.

The retarding action of ZnO and borax on cementitious systems was determined by measuring the heat of hydration of cement paste samples using a four-channel isothermal calorimeter. A 0.45 water to cementitious material ratio (w/cm) was used. When ZnO or borax was used in paste samples, they were added to dry cementitious material and mixed for one minute by hand before addition of mixing water to the sample. This was done to make sure ZnO and borax particles were distributed evenly throughout the sample. Several ZnO and borax dosages were used; all dosages are given as % mass of cementitious material.

**Figure 1:**
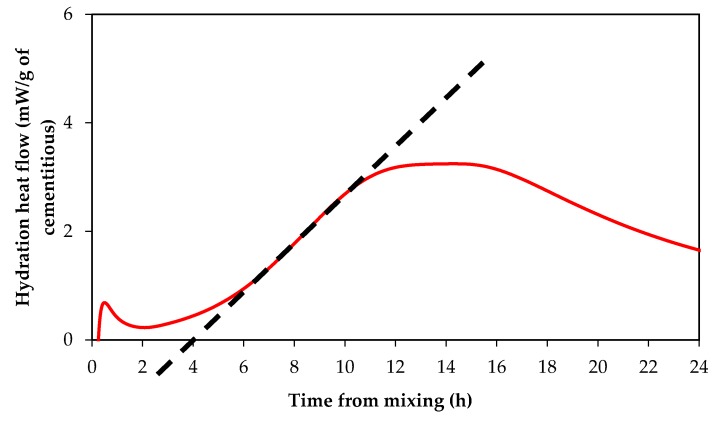
Schematic method of determining the induction period.

Paste samples were mixed with an overhead mixer at 600 rpm for 120 s, followed by a 60 s rest period, and then mixed at 600 rpm for 60 s. The mass of cement pastes samples was approximately 50 g, except for CSA samples that were approximately 15 g. For a given mix, two test samples were prepared for measuring the heat of hydration; the reported result of the heat of hydration is based on the average of the two samples. To obtain the induction period (retardation time), the slope of the acceleration peak of the heat of hydration was extended to the x-axis (time axis). The intersection point between the extended slope and x-axis was considered as the induction (dormant) period as shown in Figure 1 [7].

## 3. Results and Discussions

### 3.1. Impact of Curing Temperature on ZnO Retarding Action

**Figure 2:**
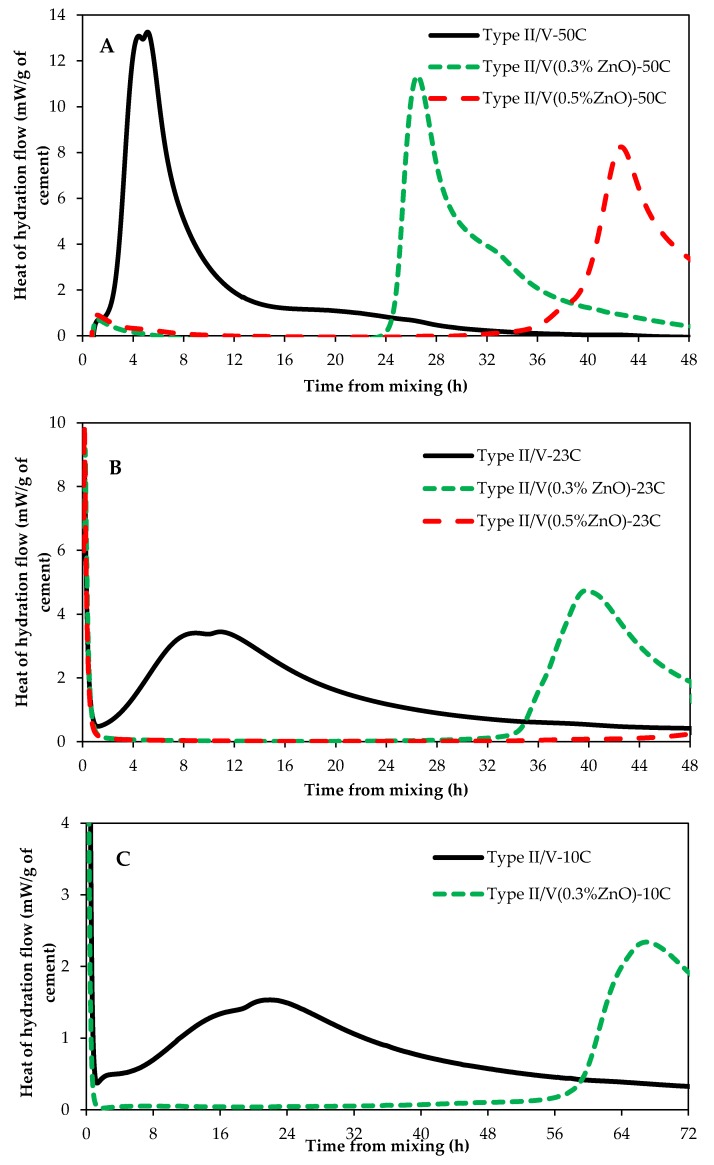
Type II/V heat of hydration at different curing temperatures; (**A**) 50 °C, (**B**) 23 °C and (**C**) 10 °C.

**Figure 3:**
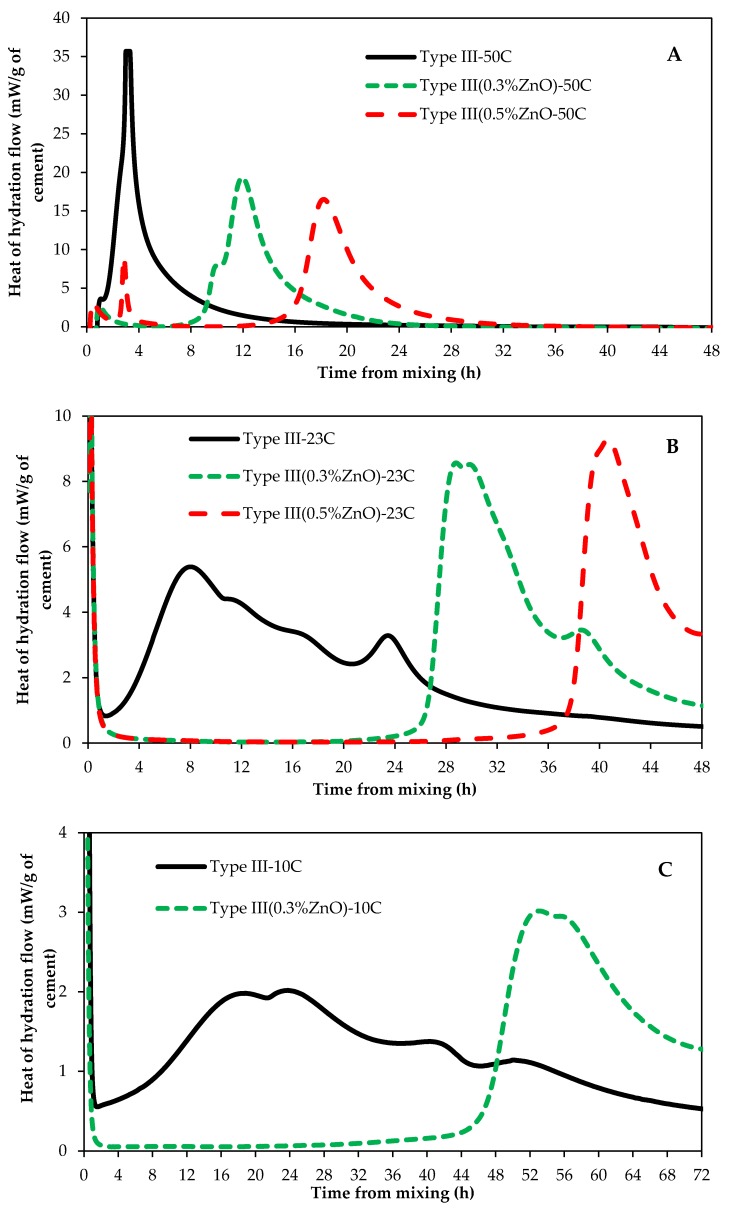
Type III heat of hydration at different curing temperatures; (**A**) 50 °C, (**B**) 23 °C and (**C**) 10 °C.

**Figure 4:**
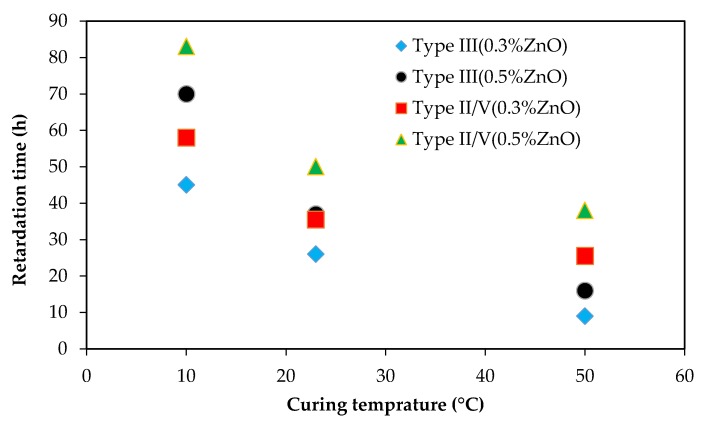
Correlation between curing temperature and retardation time.

Experiments were conducted to investigate the impact of curing temperatures on ZnO retarding action on cementitious systems. Figure 2 shows the impact of curing temperature on type II/V paste samples with and without ZnO. Figure 3 presents the influence of curing temperature on Type III samples with and without ZnO. As can be seen from these figures, the curing temperature has a huge impact on ZnO retardation action in cementitious systems; the higher the curing temperature, the shorter the retardation time. Figure 4 plots curing temperatures versus retardation time of paste samples with 0.3% ZnO and 0.5% ZnO. There seems to be an exponential correlation between curing temperature and retardation time of paste samples containing ZnO. Another interesting trend that can be seen for samples that were cured at 50 °C is that the main hydration peak height is lower from samples containing ZnO compared to the control sample (with no ZnO). This is the opposite of samples cured at 23 °C or 10 °C.

It has been proposed that the higher the curing temperature is, the higher the rate of the hydration reaction is [18]; this could mean that at higher curing temperatures, C–S–H nuclei are initiated at a faster rate. It has also been shown that higher curing temperatures increase the calcium hydroxide (CH) nucleation rate [19]. This increase in C–S–H and CH nuclei at higher curing temperatures could be the reason behind the suppressed retardation action of ZnO in cement paste, as shown in Figure 2 and Figure 3. It could be proposed that the poisoning effect of ZnO is overcome sooner at high curing temperatures compared to low curing temperatures due to an increase in C–S–H nuclei at high curing temperatures.

Comparing Figure 2 and Figure 3, it can be observed that, for a given curing temperature and ZnO dosage, type III samples had a shorter retardation time compared to those samples made with type II/V. This is because type III is finer that type II/V and this results in a higher rate of cement dissolution and a higher number of C–S–H nuclei during early stages of hydration. Therefore, the number of C–S–H nuclei in systems containing type III cement would be more than of those containing type II/V. Because of this, the poisoning ability of ZnO in systems containing type III will deplete faster than in systems with type II/V.

### 3.2. Influence of Borax on Portland Cement Hydration

**Figure 5:**
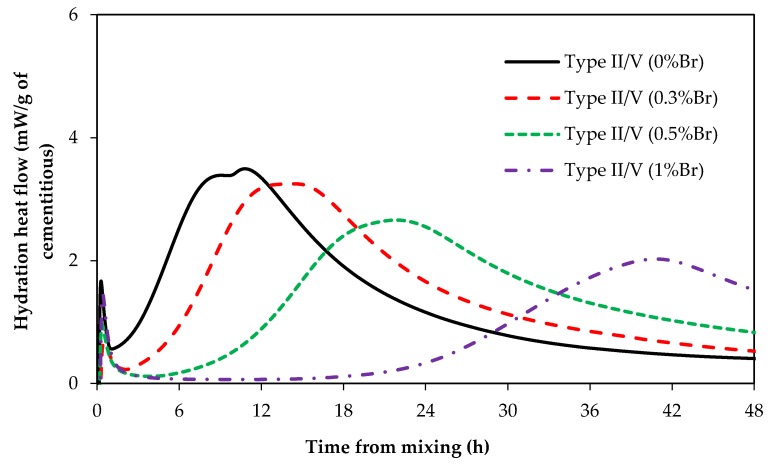
Type II/V hydration with and without borax.

**Figure 6:**
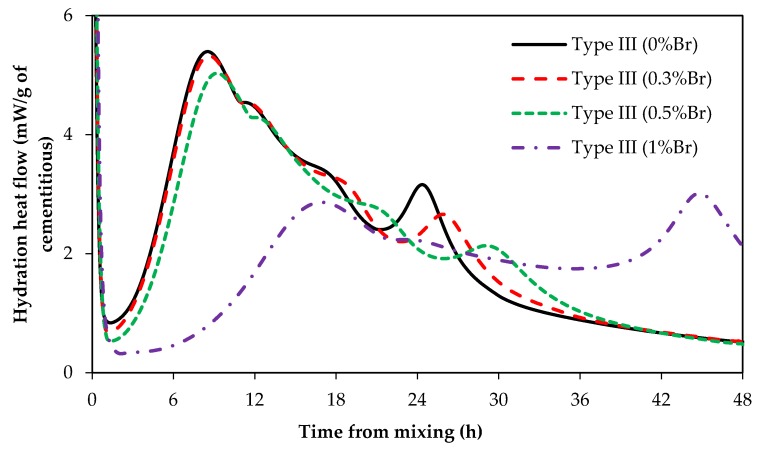
Type III hydration with and without borax.

The impact of borax on the heat of hydration of type II/V and type III cement paste is presented in Figure 5 and Figure 6. These samples were cured at 23 °C. In general, addition of borax in paste samples made with type II/V and type III delayed the set time. However, up to 0.5% dosage of borax had a negligible effect on the induction period of type III samples (refer to Figure 6).

Comparing Figure 2B with Figure 5 (or Figure 3B with Figure 6), it becomes clear that ZnO is a much stronger set time retarder than borax for Portland cement systems. Type II/V samples containing 0.5% ZnO have a retardation time of about 50 h while type II/V samples containing 0.5% borax (0.5%Br in Figure 5) have a retardation time of approximately 6 h. Similarly, type III samples containing 0.5% ZnO have a retardation time of 38 h (Figure 3B), while type III samples with 0.5% borax (0.5%Br in Figure 6) have a retardation time of about 3 h.

The other notable difference between paste samples containing borax and ZnO is that samples containing ZnO have a narrower but steep main hydration peak, whereas samples containing borax show wider but shallower main hydration peaks. The sharp narrow peaks in samples containing ZnO have been suggested to result from the high number of C–S–H nucleation sites in these samples [7]. Shallow hydration peaks in systems containing borax could suggest that borax limits dissolution of cement particles and thus reduces the number of C–S–H nuclei. The negligible effect of 0.3% and 0.5% borax on type III hydration could be attributed to the fact that type III has a high surface area, which means borax cannot reduce the cement dissolution at these lower dosages because there are more surfaces to be poisoned.

If the cement particle dissolution is reduced by borax, the number of C–S–H nuclei would be small, the rate of C–S–H formation and growth would be slower, and hence the hydration peak would be shallower. Therefore, it could be suggested that the mechanism by which ZnO and borax retard the cement set time is different; ZnO retards the set time by poisoning C–S–H nucleation and growth whereas borax prolongs the set time by poisoning/reducing cement dissolution. Borax could also poison the nucleation and/or growth of C–S–H.

### 3.3. Impact of ZnO and Borax on Calcium Aluminate Cement (CAC) Hydration

**Figure 7:**
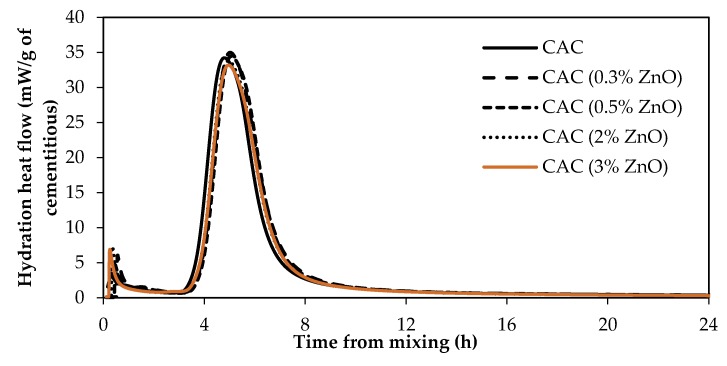
Heat of hydration of CAC with and without ZnO addition.

**Figure 8:**
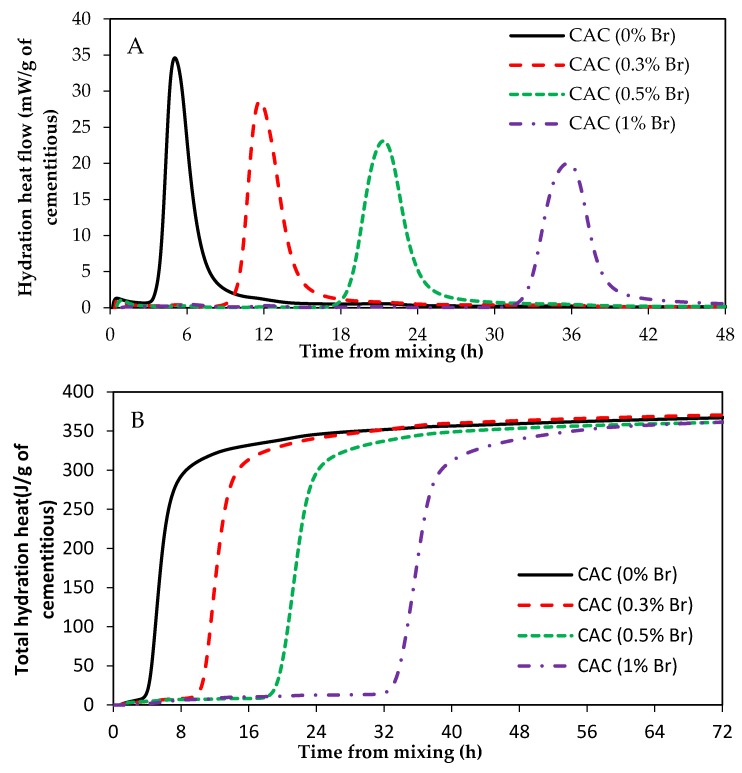
Heat of hydration of CAC with and without borax. (**A**) heat flow; (**B**) total heat.

Figure 7 shows the heat of hydration of CAC paste samples with and without ZnO. Clearly, ZnO has no retarding effect, even at high dosages, on CAC hydration. Figure 8 shows the heat of hydration of CAC paste samples containing different dosages of borax, 0%Br, 0.3%Br, 0.5%Br, and 1%Br. Figure 8A presents the heat flow and Figure 8B shows the total heat of hydration. 

As can been seen from Figure 8, addition of borax to CAC paste samples retarded the set time. Samples with 1% borax (1%Br) had an induction period of about 32 h, whereas samples with 0.3% Br had a retardation time of about 10 h. Besides prolonging the induction period, borax reduced the CAC main hydration peak height as well (refer to Figure 8A). The higher the borax dosage in the mix, the lower the peak height. However, the main hydration peaks are wider in samples containing borax compared to those without borax. Shallower but wider hydration peaks for samples containing borax suggests that borax controls the rate of nucleation and/or growth of hydration products. As it was suggested earlier, this could be because borax suppresses the dissolution of phases in cement. Furthermore, borax could also poison the nucleation and/or growth of CAC hydration products.

Hydration reactions of CAC are different from that of Portland cement. The main hydration products of CAC are calcium aluminate hydrate and aluminate hydrate. At curing temperatures between 20 °C and 30 °C, the hydration products of CAC are calcium aluminate hydrate (CAH10 and C2AH8) and aluminate hydroxide (AH3). Therefore, it can be suggested that ZnO does not poison the nucleation and/or growth of CAC hydration products, nor does ZnO reduce the dissolution of CAC phases.

### 3.4. Influence of ZnO and Borax on CSA Cement Hydration

**Figure 9:**
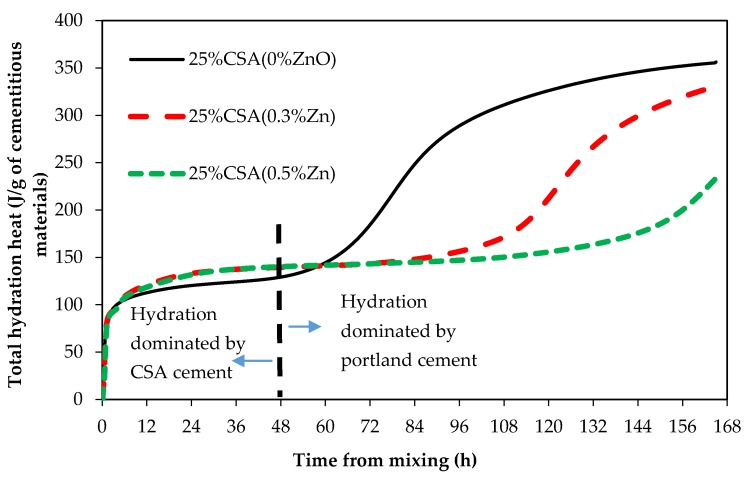
Total heat of hydration of samples containing 25% CSA.

To study the influence of ZnO addition on CSA cement, the set time of CSA cement was delayed by three methods: (1) mixing 25% CSA and 75% type II/V cements, (2) adding 50% citric acid, and (3) adding a chemical retarder. Citric acid and retarder were mixed with the mix water. Figure 9 shows the heat of hydration graphs for binary paste samples (25% CSA and 75% type II/V) with and without ZnO. 

Hydration graphs in Figure 9 show two distinct hydration phases, one from initial mixing to around 48 h, and the other one beginning at around 48 h. The hydration process of cementitious systems containing CSA and Portland cement has been proposed to be a two phase process [20]. CSA hydration happens during the first phase and produces AFm, AFt, and aluminium hydroxide (AH3). The second phase of hydration is due to the hydration of C3S in Portland cement, which forms stratlingite (C2ASH8), C–S–H, and CH according to Equations (3) and (4) [20].

(3) $$C_{3}S+AH_{3}+H\to C_{2}ASH_{8}+CH$$

(4) $$C_{3}S+H\to \text{​}CSH+CH$$

Therefore, the first phase (up to 48 h) of the hydration reaction observed in Figure 9 is dominated by CSA cement, while Portland cement hydration dominates the second phase (after 48 h). As can be seen from Figure 9, hydration of CSA was not affected by ZnO addition as the first phase of hydration showed no retardation regardless of ZnO dosage. However, hydration of type II/V (the second phase of hydration) was delayed by ZnO.

**Figure 10:**
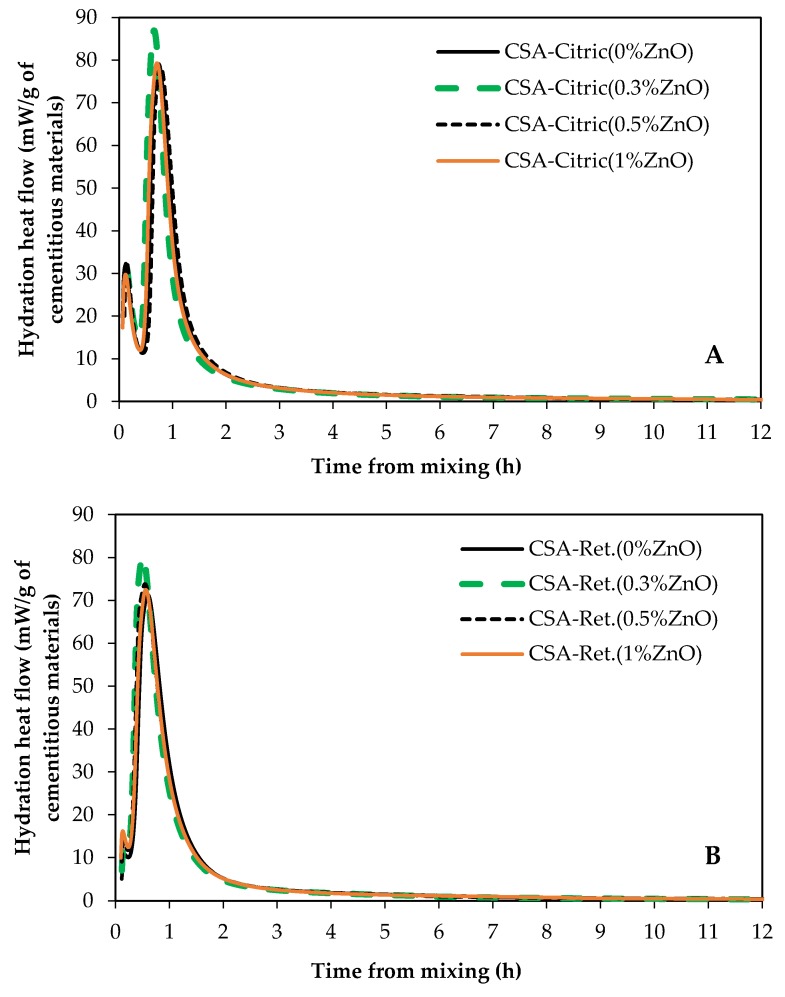
Hydration heat of CSA paste samples; (**A**) dosed by citric acid and (**B**) dosed by a chemical retarder admixture.

Figure 10A shows the heat of hydration of CSA pastes samples made with 50% citric acid solution with and without ZnO. Figure 10B shows hydration graphs for CSA cement paste samples dosed by a chemical retarder admixture (ASTM type B and D). Citric acid and retarder were used to delay the set time enough to allow sample preparation. In both Figure 10A,B, ZnO did not affect the hydration of CSA. Therefore, based on the ZnO performance in binary paste samples (Figure 8) and in 100% CSA cement paste samples (Figure 10), it can be suggested that ZnO doesn’t have a retarding effect on the CSA set time. Thus, it can be concluded that ZnO has no poisoning effect on nucleation and/or growth of CSA main hydration products (ettringite (AFt) and aluminate hydroxide (AH3)).

**Figure 11:**
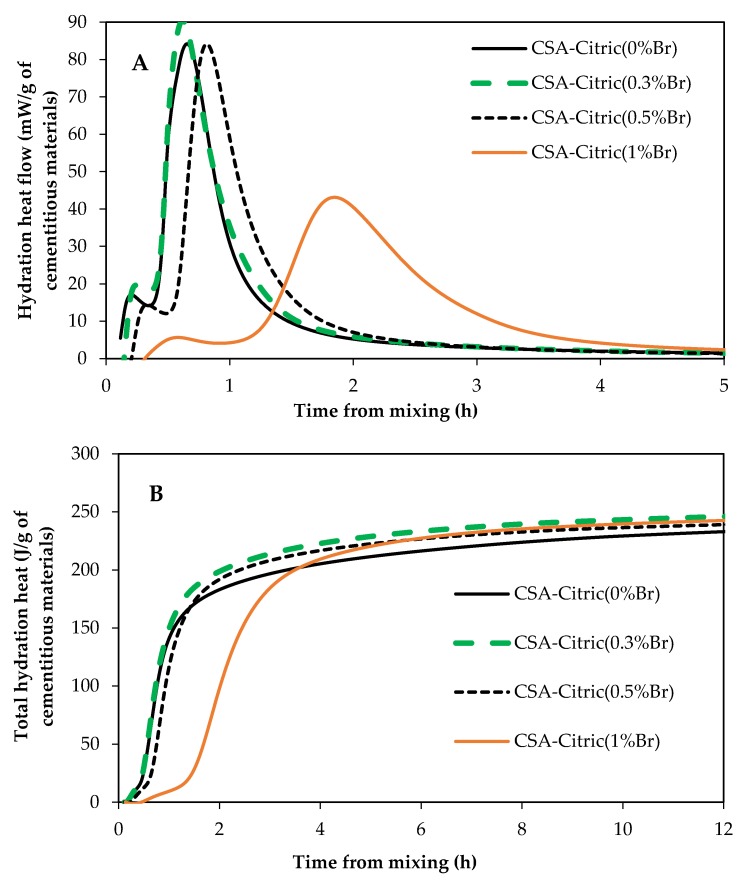
Influence of borax on CSA hydration; (**A**) heat flow and (**B**) total hydration heat.

Figure 11 shows the impact of borax on hydration of CSA paste samples mixed with 50% citric acid solution with and without borax. The sample containing 0.3% borax (0.3%Br) had the same induction period compared to the control one (0%Br). However, samples containing 0.5%Br and 1%Br had a longer induction period compared to the control sample. The induction period for the sample containing 1%Br was longer than an hour, as it can be seen in Figure 11A. Besides prolonging the induction period, borax lowered the height of the main hydration peak. However, the total heat of hydration of CSA samples at 6 h after mixing was similar regardless of the borax dosage. The retardation due to borax could be because borax prevents the dissolution of ye’elimite (C4A3Ŝ), which is a major mineral phase in CSA cement [12].

## 4. Conclusions

The retarding action of ZnO and borax on Portland cement, CSA cement, and CAC was investigated. The heat of hydration of cement paste samples with and without addition of ZnO and borax was measured to study the impact of ZnO and borax on the set time of cementitious systems. It was found that the retarding impacts of ZnO and borax on cementitious systems are different from each other. It was shown that both ZnO and borax can retard the set time of Portland cement systems; however, ZnO was found to be a stronger set time retarder than borax for Portland cement systems. However, the set time of CSA and CAC was not retarded by ZnO, while borax retarded the set time of these cementitious systems. It was also revealed that as the curing temperature raises, the effectiveness of ZnO in retarding the set time decreases.

It can be concluded that the mechanism(s) by which the set time of cementitious systems is retarded by ZnO and by borax is different. It seems like ZnO does not suppress the nucleation and growth of CSA and CAC hydration products, which are mainly aluminate bearing phases such as ettringite and aluminate hydrate. It can also be suggested that borax retards the set time by reducing the dissolution of cement particles, whereas we suggest that ZnO poisons nucleation and/or growth of C–S–H.

## Figures and Tables

**Table 1:** Oxide composition of cementitious materials.

	CSA	CAC	Type II/V	Type III
SiO_2_	8.32	4.79	21.3	21
Al_2_O_3_	21.8	40.17	3.9	5.1
Fe_2_O_3_	2.8	15.51	3.8	1.2
CaO	43.3	37.29	63.2	64.2
SO_3_	22.8	-	2	3.6
MgO	0.65	-	2.2	2.2
Blain (cm^2^/gr)	5924	4065	3800	5550
